# Relationship between Muscle Mass and Non-Alcoholic Fatty Liver Disease

**DOI:** 10.3390/biology10020122

**Published:** 2021-02-05

**Authors:** Jun-Hyuk Lee, Hye-Sun Lee, Byoung-Kwon Lee, Yu-Jin Kwon, Ji-Won Lee

**Affiliations:** 1 Department of Medicine, Graduate School of Yonsei University College of Medicine, Seoul 03722, Korea; muzzyljh@yuhs.ac; 2 Department of Family Medicine, Yonsei University College of Medicine, Yongin Severance Hospital, Yongin 16995, Korea; 3 Biostatistics Collaboration Unit, Department of Research Affairs, Yonsei University College of Medicine, Seoul 06273, Korea; HSLEE1@yuhs.ac; 4 Department of Internal Medicine, Yonsei University College of Medicine, Gangnam Severance Hospital, Seoul 06273, Korea; CARDIOBK@yuhs.ac; 5 Department of Family Medicine, Yonsei University College of Medicine, Gangnam Severance Hospital, Seoul 06273, Korea

**Keywords:** sarcopenia, non-alcoholic fatty liver disease, obesity, skeletal muscle mass, inflammation

## Abstract

**Simple Summary:**

Sarcopenia and non-alcoholic fatty liver disease share common pathological and physiological mechanisms that can co-occur with aging. Low skeletal muscle mass index and non-alcoholic fatty liver disease were related, regardless of abdominal obesity. Maintenance of muscle mass should be emphasized for prevention of non-alcoholic fatty liver disease. Management of fatty liver also could be an important strategy to preserve muscle mass.

**Abstract:**

Although sarcopenia is known to be a risk factor for non-alcoholic fatty liver disease (NAFLD), whether NAFLD is a risk factor for the development of sarcopenia is not clear. We investigated relationships between NAFLD and low skeletal muscle mass index (LSMI) using three different datasets. Participants were classified into LSMI and normal groups. LSMI was defined as a body mass index (BMI)-adjusted appendicular skeletal muscle mass <0.789 in men and <0.512 in women or as the sex-specific lowest quintile of BMI-adjusted total skeletal muscle mass. NAFLD was determined according to NAFLD liver fat score or abdominal ultrasonography. The NAFLD groups showed a higher hazard ratios (HRs) with 95% confidence intervals (CIs) for LSMI than the normal groups (HRs = 1.21, 95% CIs = 1.05–1.40). The LSMI groups also showed a higher HRs with 95% CIs for NAFLD than normal groups (HRs = 1.56, 95% CIs = 1.38–1.78). Participants with NAFLD had consistently less skeletal muscle mass over 12 years of follow-up. In conclusion, LSMI and NAFLD showed a relationship. Maintaining muscle mass should be emphasized in the management of NAFLD.

## 1. Introduction

Non-alcoholic fatty liver disease (NAFLD) has become the most common chronic liver disease worldwide with increasing obesity, metabolic syndrome, and dyslipidemia [1] affecting up to 20% in the general population [2,3] and 16–33% in Korean, respectively [4]. In addition, increased interest of the importance of NAFLD in recent years has led to studies of various treatment modalities for NAFLD [5,6,7]. However, until now, there is no firmly recommended medical treatment for NAFLD [5,6,7]. The mainstay of treatment of NAFLD is still the adoption of lifestyle modifications including sustained weight loss, increased physical activity, and maintaining the healthy weight [5,6,7].

Aging is closely associated with increased accumulation of lipids in non-adipose tissues and organs [8]. Liver is the key organ that is mainly affected by ectopic fat accumulation [9]. Because the sarcopenia and NAFLD share common pathological and physiological mechanisms, the co-occurrence of sarcopenia and NALFD has been observed in elderly [10]. Several epidemiologic studies have shown that sarcopenia was a risk factor for incident NAFLD [11,12,13,14]. However, there were few studies to investigate whether NAFLD is a risk factor or the consequential result for sarcopenia and to verify the relationship between NAFLD and sarcopenia. If NAFLD is associated with the occurrence of sarcopenia, the maintenance of muscle mass in NAFLD patients should be emphasized more strongly, as patients with sarcopenia have an increased risk of all-cause mortality [15,16]. Therefore, this study aimed to investigate the relationship between NAFLD and the skeletal muscle mass using three Korean population-based datasets.

## 2. Results

### 2.1. General Characteristics of the Study Population

The clinical characteristics of the study population according to NAFLD status are shown in Table 1. According to the analysis of study population, by classifying them into normal and NAFLD groups, the proportions of male sex were significantly higher in the NAFLD group than that in the normal group in all three datasets. The mean levels of age, waist circumference (WC); body mass index (BMI); mean blood pressure (MBP); total cholesterol, triglyceride, aspartate aminotransferase (AST); and alanine aminotransferase (ALT) were significantly higher in the NAFLD group than those in the normal group. The mean value of basal energy expenditure (BEE) and high-density lipoprotein (HDL) cholesterol were significantly lower in the NAFLD group. Prevalence of abdominal obesity and proportion of history of cardiovascular disease (CVD) were also higher in the NAFLD group, whereas proportion of current smoker was higher in the NAFLD group. The proportion of current drinker regular exercise was higher in the NAFLD group from the Gangnam Severance Hospital Check-up (GSHC) dataset, whereas those were higher in the NAFLD group from Korean National Health and Nutrition Examination Survey (KNHANES) and Korean Genome and Epidemiology Study (KoGES) dataset. The NAFLD group in the KoGES had significantly higher daily amount of total caloric intake, and carbohydrate intake although the NAFLD group in the KNHANES did not show significant differences in all nutrients intake. The mean value of skeletal muscle mass index (SMI) was lower in the NAFLD groups in all dataset. The proportions of LSMI were significantly higher in NAFLD group than normal group.

The clinical characteristics of the study population according to presence of low skeletal muscle maa index (LSMI) are shown in Table 2. The mean levels of age, WC, BMI, MBP, total cholesterol, triglyceride, low-density lipoprotein (LDL) cholesterol, AST, and ALT were significantly higher in the LSMI group than those in the normal group from the all datasets. The mean values of BEE, HDL cholesterol, daily caloric intake, protein intake, and fat intake were lower in the LSMI group. The mean value of NAFLD-liver fat score and prevalence of NAFLD were higher in the LSMI group in the KNHANES and KoGES.

**Figure 1:**
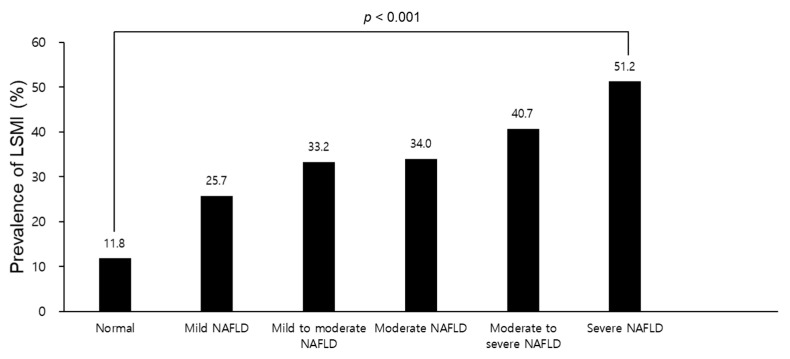
Prevalence of LSMI according to the grade of fatty liver examined by abdominal ultrasonography in the GSHC. Abbreviations: LSMI, low skeletal muscle mass index; GSHC, Gangnam Severance Hospital Check-up; NAFLD, non-alcoholic fatty liver disease.

Figure 1 presents prevalence of LSMI according to grade of fatty liver examined by abdominal ultrasonography in the GSHC. Compared to normal (11.8%), prevalence of LSMI gradually increased with increasing fatty liver grade (*p* < 0.001).

### 2.2. Relationship between SMI and NAFLD

In the first cohort set of the KoGES, the incident LSMI rate per 2 years were ranged from 2.0 to 19.9. Moreover, incident NAFLD rate per 2 years were ranged from 4.6 to 10.4 in the analysis of the second cohort set of the KoGES (Table S1). The cumulative incidence of LSMI according to NAFLD status in the KoGES data is presented in Figure S1 as Kaplan–Meier curves. NAFLD group had higher cumulative incidence of LSMI over 12 years with significance (log-rank test, *p* < 0.001). Figure S2 shows Kaplan–Meier curves for cumulative incident NAFLD according to LSMI status. LSMI group showed higher cumulative incidence of NAFLD over 12 years with significance (log-rank test, *p* < 0.001).

**Figure 2:**
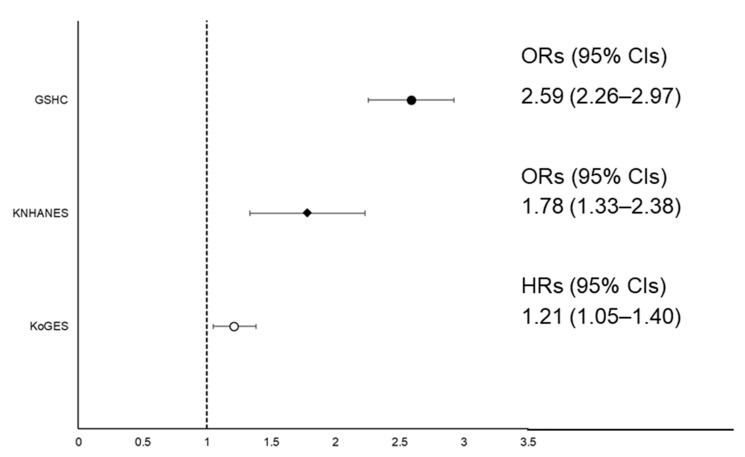
Forest plot of ORs or HRs with 95% CIs for low skeletal muscle mass index (LSMI) according to NAFLD status. Abbreviations: HRs, hazard ratios; ORs, odds ratios; CIs, confidence intervals; LSMI, low skeletal muscle mass index; NAFLD, non-alcoholic fatty liver disease; KNHANES, Korean National Health and Nutrition Examination Survey; KoGES, Korean Genome and Epidemiology Study.

**Figure 3:**
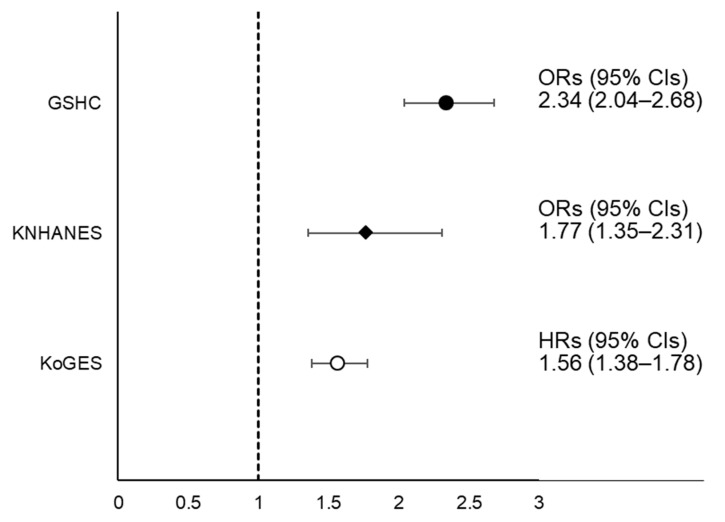
Forest plot of ORs or HRs with 95% CIs for NAFLD according to LSMI status. Abbreviations: HRs, hazard ratios; ORs, odds ratios; CIs, confidence intervals; LSMI, low skeletal muscle mass index; NAFLD, non-alcoholic fatty liver disease; KNHANES, Korean National Health and Nutrition Examination Survey; KoGES, Korean Genome and Epidemiology Study.

Figure 2 and Figure 3 show the association between SMI and NAFLD in the three datasets. In Figure 2, the hazard ratios (HRs) with 95% confidence intervals (CIs) for incident LSMI in the NAFLD group in the KoGES, compared to normal group, was 1.21 (1.05–1.40) after adjusting for age, sex, abdominal obesity, physical activity, smoking status, current drinking status, BEE, daily protein intake, MBP, fasting glucose, total cholesterol, and history of CVD. In the subgroup analysis, the high energy intake/BEE ratio (EI/BEE) subgroup and physically inactive group had significantly higher HRs with 95% CIs for incident LSMI of the NAFLD group compared to the normal group (Table S2). The fully adjusted HRs (95% CIs) for incident LSMI per 1 standard deviation (SD) increase in NAFLD-liver fat score are shown in Table S3. The fully adjusted odds ratios (ORs) (95% CIs) incident LSMI in the NAFLD group were 1.78 (1.33–2.38) in the KNHANES and 2.59 (2.26–2.97) in the GSHC, respectively, after adjusting all confounding variables, except for daily protein intake in the GSHC.

In Figure 3, the fully adjusted HR (95% CIs) for incident NAFLD in the LSMI group in the KoGES, compared to normal group, was 1.56 (1.38–1.78). In the subgroup analysis, the HR with 95% CI for incident NAFLD of LSMI was significantly higher compared to normal SMI regardless of EI/BEE and physical activity status (Table S4). The fully adjusted HRs (95% CIs) of incident NAFLD per sex-specific 1 SD increase in SMI are shown in Table S5. The fully adjusted ORs (95% CIs) for incident NAFLD in LSMI groups were 1.77 (1.35–2.31) in the KNHANES and 2.34 (2.04–2.68) in the GSHC.

Table 3 shows the independent association among LSMI status with or without abdominal obesity and NAFLD. The fully adjusted HRs (95% CIs) for incident NAFLD in the LSMI without abdominal obesity group, normal SMI with abdominal obesity group, and LSMI with abdominal obesity group in the KoGES, compared to normal SMI without abdominal obesity group, were 1.57 (1.35–1.83), 1.39 (1.18–1.65), and 2.15 (1.79–2.60), respectively, after adjusting for all confounding variables, except for abdominal obesity. The fully adjusted ORs (95% CIs) for NAFLD in the LSMI without abdominal obesity group, normal SMI with abdominal obesity, and LSMI with abdominal obesity, compared to normal SMI without abdominal obesity group, were 1.67 (1.08–2.56), 1.42 (1.10–1.83), and 2.63 (1.87–3.71) in the KNHANES and 2.76 (2.34–3.27), 3.14 (2.70–3.66), and 5.67 (4.80–6.69) in the GSHC, respectively.

### 2.3. Longitudinal Changes in SMI According to NAFLD

**Figure 4:**
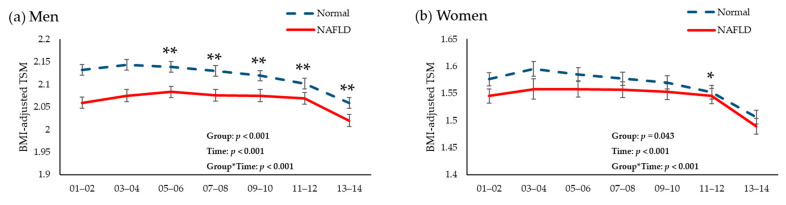
Changes in total skeletal muscle mass/body mass index during the 12-year follow-up period according to the presence of non-alcoholic fatty liver disease in (**a**) men and (**b**) women. Abbreviations: NALFD, non-alcoholic fatty liver disease; BMI, body mass index; TSM, total skeletal muscle mass index; CVD, cardiovascular disease. *p* was calculated after adjusting for age, sex, abdominal obesity, physical activity, smoking status, current drinking status, basal energy expenditure, daily protein intake, mean blood pressure, fasting glucose, total cholesterol, and history of CVD. * *p* < 0.05. ** *p* < 0.001.

Figure 4 presents the longitudinal changes in mean values of SMI over 12 years of follow-up period to baseline NAFLD status in men and women from the KoGES data. The mean value of SMI was significantly lower in the NAFLD group than in the normal group during the follow-up period, although mean values of SMI at baseline survey were not significantly different between groups.

## 3. Discussion

In this longitudinal cohort study, we documented a relationship between LSMI and NAFLD regardless of abdominal obesity. Participants with NAFLD consistently had lower mean SMI values than those without NAFLD during all follow-up periods. In the subgroup analysis, the HRs for incident LSMI was significantly higher in the physically inactive subgroup, which emphasizes the importance of physical activity. On the other hand, the HRs for incident NAFLD was increased regardless of EI/BEE or physical activity, which suggests LSMI may be a more important risk factor than diet and exercise in the development of NAFLD.

In most guidelines, the management of NAFLD has primarily involved efforts to reduce oxidative stress and to improve insulin resistance, along with weight loss, for the prevention and treatment of NAFLD [5,6,7]. Recently, however, emerging studies have suggested the importance of maintaining and increasing muscle mass for preventing NAFLD [11,12,13,14]. In line with previous studies, we verified that participants who had LSMI were more likely to have incident NAFLD. Furthermore, we confirmed that both LSMI with and without abdominal obesity were significantly related with an increased risk for incident NAFLD. Therefore, maintaining adequate muscle mass could be an effective strategy through which to reduce the risk of NAFLD independently of abdominal obesity. Interestingly, although normal SMI with abdominal obesity group showed higher ORs or HRs with 95% CIs compared to LSMI without abdominal obesity group in the unadjusted model in all three datasets, the ORs or HRs with 95% CIs were higher in the LSMI without abdominal obesity group than in the normal SMI with abdominal obesity group in the fully adjusted model in the KNHANES and KoGES. As there was no consistent result for each dataset, it was difficult to verify the superiority between muscle mass and abdominal obesity on the effect of incident NAFLD.

There have been limited studies on the association between NAFLD and incident LSMI [17,18,19]. We discovered that participants with NAFLD were more likely to develop LSMI after adjusting for abdominal obesity and other confounding factors using three different datasets. In addition, to the best of our knowledge, this is the first study to consider changes in muscle mass according to the presence of NAFLD. We found that mean values of SMI were consistently lower in NAFLD patients than in normal individuals during 12 years of follow-up. Previous studies reported that hepatic disorders may affect muscle protein homeostasis via insulin resistance, chronic inflammation, and decreased myokines [17,20]. In this study, however, the difference of SMI between groups gradually decreased over time, suggesting that the cross-sectional association might be the driving factor for incident low muscle mass. In addition, because we set SMI as BMI-adjusted TSM in the KoGES, the gap of SMI between groups could be narrowed over time if BMI-related factors including exercise, diet, and body composition were more effective than NAFLD on muscle metabolism. Therefore, it is inconclusive whether NAFLD aggravates muscle degradation in this study. Further researches should be warranted to verify how much NAFLD affects the muscle mass.

Although the precise mechanism of the relationship between SMI and NAFLD cannot be defined by our results, we suggest a possible explanation between muscle and liver metabolism via hepatokines and myokines. Hepatokines, including leukocyte cell-derived chemotaxin 2 (LECT2) and hepassocin (HSP), can contribute to the development of LSMI by increasing insulin resistance [21,22]. In experimental models, LECT2 has been found to impair insulin signaling pathway through phosphorylation of Jun NH2-terminal kinase in C2C12 myocyte [21] and HSP has been shown to contribute to the development of insulin resistance in skeletal muscle via epidermal growth factor receptor/c-Jun N-terminal kinase (EGFR/JNK)-mediated pathway [22]. Irisin, a myokine that promotes energy expenditure [23], has also been shown to improve hepatic steatosis by activating AMP-activated protein kinase and by inhibiting transcription of sterol regulatory element-binding transcription factor 2 in hepatocytes [24]. Therefore, reduced irisin secretion with a decrease in muscle mass might affect incident NAFLD.

Our study has several limitations. First, we could not evaluate various risk factors associated with fatty liver, such as autoimmune hepatitis, Wilson’s disease, and medications, due to a lack of information. However, autoimmune hepatitis and Wilson’s disease are rare diseases, and we excluded viral hepatitis and habitual heavy drinking to minimize confounding factors between muscle mass and NAFLD, a major cause of chronic liver disease in Koreans [25]. Second, as we could not use ASM in the GSHC and KoGES due to the lack of data, we could not apply the commonly used diagnostic criteria for sarcopenia. To overcome this limitation, we also performed analysis using the KNHANES dataset with ASM data by applying the Foundation for the National Institutes of Health (FNIH) Sarcopenia Project criteria. In addition, although the definition of sarcopenia includes not only muscle mass but also muscle strength and performance, we could not evaluate muscle strength or performance. Further studies considering muscle strength and performance should be conducted. Finally, our results would be difficult to apply to other races and await further validation in more diverse populations.

## 4. Materials and Methods

### 4.1. Study Population

This study consists of three different datasets: The KoGES, the 2008–2010 KNHANES, and the GSHC.

The KoGES is a longitudinal cohort study designed to identify risk factors for non-communicable diseases [26]. At the baseline survey conducted in 2001–2002, adults aged 40–69 years in urban (Ansan) and rural areas (Ansung) were recruited into the cohort. The participants were biennially followed up in the study until 2013–2014. The KNHANES is a nationwide, representative, population-based survey annually conducted by the Korea Centers for Disease Control and Prevention. Sample weights were assigned to participants to represent the general Korean population. At the GSHC, adults underwent a medical examination between 1 October 2016 and 31 January 2017.

The study population selection processes are described in Figure S3. From a total of 10,030 participants in the KoGES baseline survey, we selected a total of 6567 participants by applying common exclusion criteria: (1) those who had a history of hepatitis (*n* = 423), (2) those who consumed ≥30 g/day of alcohol in men and ≥20 g/day in women (*n* = 964), (3) those for whom NAFLD liver fat score could not be calculated (*n* = 276), and (4) those without bioelectrical impedance analysis (BIA) data (*n* = 1800). In the first cohort analysis, we included a total of 4587 participants to analyze incident low skeletal muscle mass index (LSMI) according to NAFLD status after further excluding those who had LSMI at the baseline survey (*n* = 1313) and those who had no follow-up data from the baseline survey (*n* = 667). In the second cohort analysis to confirm the relationship between SMI and incident NAFLD, a total of 4236 participants was finally included after further excluding those who had NAFLD at baseline survey (*n* = 1677) and those who had been lost follow-up thereafter (*n* = 654).

In the 2008–2010 KNHANES database, from a total of 21,811 adults, we excluded those who were positive for HBsAg (*n* = 1341), who were positive for anti-HCV antibody (*n* = 12), those who consumed ≥30 g/day of alcohol in men and ≥20 g/day in women (*n* = 1791), those for whom NAFLD liver fat score could not be calculated (*n* = 8390), and those missing dual energy X-ray absorptiometry (DXA) data (*n* = 1957). Finally, 8320 individuals were included in the analysis.

In the GSHC database, from a total of 19,710 adults, we excluded those who were positive for HBsAg (*n* = 1208), those who were positive for anti-HCV antibody (*n* = 951), those who consumed ≥30 g/day of alcohol in men and ≥20 g/day in women (*n* = 793), and those without abdominal ultrasonography data (*n* = 17) or BIA data (*n* = 8485). Finally, a total of 9691 participants was included in the analysis.

Informed consent was obtained from all participants in the KoGES, KNHANES, and GSHC. This study was approved by the Institutional Review Boards (IRB) of Yongin Severance Hospital (IRB number: 9-2020-0043) and Gangnam Severance Hospital (IRB number: 3-2019-0135).

### 4.2. Assessment of Body Composition

Body weight and height were measured to the nearest 0.1 kg and 0.001 m, respectively. BMI was calculated as body weight divided by height squared (kg/m^2^). WC was measured in the horizontal plane midway between lowest rib and the iliac crest. We defined abdominal obesity as WC ≥ 90 cm in men and ≥85 cm in women according to Korean specific cut-offs for abdominal obesity [27].

Participants from the three datasets, respectively, were classified into the LSMI group or normal group. In the GSHC and KoGES, total skeletal muscle mass (TSM) values were obtained from a multi-frequency BIA (Inbody 330, Biospace, Seoul, Korea), which has been used to assess sarcopenia [28,29]. In the 2008–2010 KNHANES, body composition was assessed by DXA examinations (QDR 4500A; Hologic Inc., Bedford, MA, USA). Body composition data were collected for the head, trunk, pelvic region, arms, legs, and whole body. Skeletal muscle mass was calculated as follows: lean body mass (g)—bone mineral content (g). We calculated appendicular skeletal muscle mass (ASM) using the sum of skeletal muscle mass values for both the arms and legs.

Combining the height-adjusted and weight-adjusted skeletal muscle indices was more closely associated with poor physical performance than using either of the indices alone [30]. Therefore, we defined SMI as TSM (kg)/BMI in the GSHC and KoGES and as ASM (kg)/BMI in the KNHANES. Finally, LSMI was defined as the sex-specific lowest quintile of SMI in the GSHC and the KoGES and as a SMI < 0.789 in men and <0.512 in women according to the FNIH Sarcopenia Project criteria in the KNHANES [31].

In addition, we further analyzed four groups (LSMI without abdominal obesity, normal SMI with abdominal obesity, LSMI with abdominal obesity, and normal SMI without abdominal obesity) to investigate the relationship between SMI and NAFLD in consideration of abdominal obesity.

### 4.3. Assessment of NAFLD

In the GSHC, NAFLD was diagnosed if fatty liver, focal fat sparing, or fat deposition was observed on abdominal ultrasonography performed by a well-trained radiologist. Fatty liver was classified into five categories: mild, mild to moderate, moderate, moderate to severe, or severe. In the KNHANES and the KoGES, we defined NAFLD using a validated fatty liver prediction model, NAFLD-liver fat score [32]. The calculation equation is described in Table S6. Finally, participants in the three datasets were classified into two groups: NAFLD group and normal group.

### 4.4. Covariates

In all three datasets, systolic blood pressure (SBP) and diastolic blood pressure (DBP) were defined as the average of the last two of three measured values. MBP was then calculated as DBP + 1/3 ∗ (SBP − DBP). Blood tests for measuring serum insulin, total cholesterol, triglyceride, HDL cholesterol, LDL cholesterol, AST, ALT, and plasma glucose levels were performed after at least 8 h of fasting using a Hitachi 700-110 Chemistry Analyzer (Hitachi Co., Tokyo, Japan). Smoking and drinking status were reported via self-reported questionnaires. We divided smoking status into two categories: current smoking or not. We also divided alcohol drinking status into two categories: current drinking or not. The equation used to calculate BEE is described in Table S7 [33]. Participants were classified into two categories according to the sex-specific median values of EI/BEE (1.23 in men and 1.43 in women, respectively): high EI/BEE group and low EI/BEE group [34]. Participants who had experienced ischemic stroke, myocardial infarction, or angina pectoris were considered to have a history of cardiovascular disease (CVD). Physical activity was divided into two categories: regular exercise and irregular exercise. Regular exercise was defined as exercising ≥3 times/week in the KoGES and the GSHC, whereas regular exercise was defined when a person exercises vigorously ≥20 min at least 3 days/week or ≥30 min of moderate exercise/walking at least 5 days/week in the KNHANES.

For the KoGES, a validated 103-food item food frequency questionnaire (FFQ) was used. In the KNHANES, well-trained dietitians conducted in-person interviews with participants for dietary surveillance using 24-h recall methods. We used daily total calorie intake (kcal/day), protein intake (g/day), fat intake (g/day), and carbohydrate intake (g/day) calculated through 24-h recall methods in KNHANES and FFQ in the KoGES, respectively. In the GSHC, nutritional status was not recorded.

### 4.5. Statistical Analysis

All data in the KoGES and the GSHC are presented as means ± SD or medians (25th, 75th quartile) for continuous variables and as percentage (%) for categorical variables. For continuous variables, independent t-tests were used to compare differences between two groups. For categorical variables, chi-square tests were used to compare differences between groups. All data in the 2008–2010 KNHANES are presented as mean ± standard error (SE) for continuous variables and as percentage (%) for categorical variables. For continuous variables, weighted independent t-tests were used to compare differences between groups. Weighted chi-square tests were used to compare differences between groups for categorical variables.

In the KoGES, cumulative incidence of LSMI and NAFLD were represented by Kaplan–Meier curves. We used log-rank tests to determine if distributions of cumulative incident LSMI and NAFLD differed between groups. Multivariate Cox proportional hazards regression models were used to calculate HRs with 95% CIs for incident LSMI according to NAFLD after adjusting for potential confounding variables. Conversely, HRs with 95% CIs for development of NAFLD in the LSMI group in comparison to the normal SMI group were calculated through multivariate Cox proportional hazards regression models. Subgroup analyses by EI/BEE and physical activity were performed. We also calculated HRs with 95% CIs for incident NALFD according to SMI considering abdominal obesity using multivariate Cox proportional regression models. A linear mixed model for repeated measures was used to assess the longitudinal relationship between baseline NAFLD status and subsequent changes in BMI-adjusted total skeletal muscle mass over 12 years of follow-up after adjusting for baseline confounding factors. In the KNHANES and the GSHC, ORs and 95% CIs were calculated using a multivariate logistic regression analysis to evaluate the relationship between SMI and NAFLD.

Statistical analyses were conducted using SAS statistical software (version 9.4; SAS Institute Inc., Cary, NC, USA) in the KoGES and SPSS statistical software (version 23.0; SPSS Inc., Chicago, IL, USA) in the GSHC and the KNHANES. The significance level was set at *p* < 0.05.

## 5. Conclusions

LSMI and NAFLD exhibit a relationship regardless of abdominal obesity. Furthermore, participants with NAFLD consistently had lower muscle mass than those without. Thus, strategies to preserve muscle mass would be helpful to prevent NAFLD. Furthermore, lifestyle modification to decrease NAFLD could be helpful to inhibit muscle loss. Experimental studies are needed to identify the underlying mechanism between muscle mass and hepatic steatosis.

## Figures and Tables

**Table 1:** Clinical characteristics of three different population cohorts according to non-alcoholic fatty liver disease (NAFLD) status.

	2016–2019 Gangnam Severance Hospital Check-Up			2008–2010 KNHANES			KoGES: Ansan-Ansung Study		
Variables	Normal	NAFLD	*p* *	Normal	NAFLD	*p* ^†^	Normal	NAFLD	*p* *
N	5523	4168		6343	1977		3579	1008	
Male sex, %	37.6	65.5	<0.001	37.9	49.6	<0.001	40.9	47.2	<0.001
Age, years	47.4 ± 13.1	51.2 ± 11.5	<0.001	47.3 ± 0.4	51.0 ± 0.6	<0.001	50.0 ± 8.3	52.3 ± 8.4	<0.001
Abdominal obesity, %	11.4	47.6	<0.001	17.5	45.3	<0.001	13.0	55.2	<0.001
Waist circumference, cm	76.7 ± 9.3	88.2 ± 9.6	<0.001	78.6 ± 0.2	87.0 ± 0.3	<0.001	78.7 ± 7.7	87.7 ± 7.6	<0.001
BMI, kg/m^2^	22.5 ± 3.0	26.1 ± 3.5	<0.001	22.9 ± 0.1	25.6 ± 0.1	<0.001	23.5 ± 2.6	26.0 ± 2.7	<0.001
Mean blood pressure, mmHg	86.6 ± 8.8	89.7 ± 9.2	<0.001	89.6 ± 0.3	94.5 ± 0.4	<0.001	89.8 ± 12.2	98.5 ± 11.7	<0.001
Basal energy expenditure, kcal/day	1340.9 ± 209.1	1484.2 ± 227.3	<0.001	1333.3 ± 3.5	1421.8 ± 7.6	<0.001	1356.4 ± 179.1	1452.4 ± 207.9	<0.001
Fasting glucose, mg/dL	94.5 ± 13.7	106.7 ± 25.9	<0.001	93.8 ± 0.3	109.0 ± 1.0	<0.001	82.5 ± 11.9	97.4 ± 32.3	<0.001
Fasting insulin, μIU/mL	-	-	-	8.5 ± 0.1	14.1 ± 0.2	<0.001	6.5 ± 2.7	10.7 ± 7.7	<0.001
Total cholesterol, mg/dL	201.8 ± 36.3	208.3 ± 41.2	<0.001	185.6 ± 0.6	193.4 ± 1.2	<0.001	187.3 ± 33.6	201.0 ± 36.4	<0.001
Triglyceride, mg/dL	88 (67, 120)	141 (102, 197)	<0.001	115.1 ± 1.4	172.6 ± 4.0	<0.001	115 (89, 151)	191 (150, 258)	<0.001
HDL cholesterol, mg/dL	61.1 ± 13.3	51.6 ± 11.3	<0.001	49.3 ± 0.2	44.2 ± 0.3	<0.001	46.3 ± 9.9	39.6 ± 7.9	<0.001
LDL cholesterol, mg/dL	126.0 ± 29.6	135.7 ± 32.6	<0.001	113.4 ± 0.6	114.7 ± 1.1	0.306	115.1 ± 30.4	119.8 ± 33.0	<0.001
AST, IU/L	24 (20, 30)	27 (22, 35)	<0.001	19.8 ± 0.1	29.9 ± 0.8	<0.001	25 (22, 29)	29 (24, 36)	<0.001
ALT, IU/L	18 (13, 25)	29 (20, 41)	<0.001	17.2 ± 0.1	36.2 ± 0.8	<0.001	20 (16, 26)	31 (24, 45)	<0.001
Current smoker, %	13.3	22.7	<0.001	18.7	22.7	0.008	21.0	24.0	0.045
Current drinker, %	60.9	63.9	0.002	52.3	51.1	0.474	44.6	42.3	0.195
Regular exercise, %	23.8	25.6	0.043	23.6	20.4	0.028	52.1	49.8	0.193
Daily caloric intake, kcal/day	-	-	-	1855.5 ± 14.9	1889.4 ± 27.4	0.225	1842.8 (1539.9, 2194.0)	1881.1 (1577.5, 2291.0)	0.003
Daily protein intake, g/day	-	-	-	67.0 ± 0.8	68.8 ± 1.2	0.171	62.4 (48.4, 78.0)	63.9 (50.3, 79.8)	0.057
Daily fat intake, g/day	-	-	-	36.4 ± 0.6	37.5 ± 1.2	0.323	29.9 (20.1, 41.1)	29.8 (19.7, 41.7)	0.579
Daily CHO intake, g/day	-	-	-	308.2 ± 2.4	312.1 ± 4.2	0.380	322.7 (277.8, 375.1)	331.0 (288.1, 397.2)	<0.001
Skeletal muscle index	1.866 ± 0.352	1.845 ± 0.356	0.005	0.751 ± 0.003	0.744 ± 0.006	0.308	1.828 ± 0.312	1.794 ± 0.305	0.002
NAFLD liver fat score	-	-	-	−1.819 ± 0.127	0.368 ± 0.036	<0.001	−1.951 ± 0.638	0.479 ± 1.606	<0.001
LSMI, %	11.8	30.9	<0.001	8.4	15.7	<0.001	-	-	-
History of CVD, %	4.4	5.7	0.002	3.0	5.2	0.001	2.1	3.3	0.046

Abbreviations: KNHANES, Korean National Health and Nutrition Examination Survey; KoGES, The Korean Genome and Epidemiology Study; NAFLD, non-alcoholic fatty liver disease; SE, standard error; BMI, body mass index; HDL, high-density lipoprotein; LDL, low-density lipoprotein; AST, aspartate aminotransferase; ALT, alanine aminotransferase; CHO, carbohydrate; LSMI, low skeletal muscle mass index; CVD, cardiovascular disease. * *p* derived from Student’s t-test for continuous variables and chi-square test for categorical variables. ^†^ *p* derived from weighted generalized linear regression analysis for continuous variables and weighted chi-square test for categorical variables.

**Table 2:** Clinical characteristics of three different population cohorts according to skeletal muscle mass index (SMI) status.

	2016–2019 Gangnam Severance Hospital Check-Up			2008–2010 KNHANES			KoGES: Ansan-Ansung Study		
Variables	Normal	LSMI	*p* *	Normal	LSMI	*p* ^†^	Normal	LSMI	*p* *
N	7753	1938		7412	908		3405	831	
Male sex, %	49.6	49.7	0.939	40.3	41.9	0.487	41.7	42.8	0.562
Age, years	47.5 ± 12.1	55.5 ± 12.3	<0.001	45.6 ± 0.4	62.0 ± 0.8	<0.001	49.9 ± 8.3	55.1 ± 8.8	<0.001
Abdominal obesity, %	19.3	57.9	<0.001	21.1	47.3	<0.001	12.2	30.3	<0.001
Waist circumference, cm	79.7 ± 10.2	89.4 ± 10.7	<0.001	79.7 ± 0.2	86.8 ± 0.6	<0.001	78.6 ± 7.6	83.8 ± 8.0	<0.001
BMI, kg/m^2^	23.2 ± 3.1	27.6 ± 3.9	<0.001	23.3 ± 0.1	25.8 ± 0.2	<0.001	23.4 ± 2.5	25.9 ± 3.0	<0.001
Mean blood pressure, mmHg	87.5 ± 8.9	90.0 ± 9.4	<0.001	90.1 ± 0.3	95.6 ± 0.6	<0.001	89.8 ± 12.2	94.1 ± 12.6	<0.001
Basal energy expenditure, kcal/day	1413.7 ± 229.8	1357.8 ± 217.3	<0.001	1362.4 ± 3.7	1268.3 ± 7.4	<0.001	1359.2 ± 179.8	1314.1 ± 167.0	<0.001
Fasting glucose, mg/dL	97.9 ± 18.6	107.1 ± 26.7	<0.001	96.3 ± 0.3	105.2 ± 1.3	<0.001	82.4 ± 11.6	83.3 ± 12.7	0.057
Fasting insulin, μIU/mL	-	-	-	9.6 ± 0.1	11.1 ± 0.3	<0.001	6.4 ± 2.7	6.5 ± 2.8	0.914
Total cholesterol, mg/dL	203.5 ± 37.8	208.9 ± 41.4	<0.001	186.4 ± 0.6	196.0 ± 1.8	<0.001	186.9 ± 33.7	195.9 ± 34.1	<0.001
Triglyceride, mg/dL	102 (73, 146)	130 (94, 181)	<0.001	123.9 ± 1.5	163.1 ± 4.9	<0.001	115 (89, 150)	133 (102, 179)	<0.001
HDL-cholesterol, mg/dL	57.9 ± 13.5	53.4 ± 11.9	<0.001	48.4 ± 0.2	45.4 ± 0.5	<0.001	46.3 ± 10.0	45.1 ± 9.8	<0.001
LDL-cholesterol, mg/dL	128.9 ± 30.7	135.4 ± 33.2	<0.001	113.2 ± 0.5	118.0 ± 1.7	0.007	114.7 ± 30.4	121.2 ± 31.8	<0.001
AST, IU/L	24 (20, 31)	28 (23, 36)	<0.001	21.8 ± 0.2	23.9 ± 0.5	<0.001	25 (22, 29)	26 (23, 30)	<0.001
ALT, IU/L	20 (14, 30)	27 (19, 41)	<0.001	21.1 ± 0.3	23.7 ± 0.8	0.002	19 (16, 26)	21 (18, 28)	<0.001
Current smoker, %	17.7	15.7	0.038	19.9	16.2	0.091	21.4	17.4	0.011
Current drinker, %	64.6	52.4	<0.001	53.2	41.6	<0.001	45.0	39.0	0.002
Regular exercise, %	23.7	28.0	<0.001	23.3	19.0	0.049	52.2	48.7	0.071
Daily caloric intake, kcal/day	-	-	-	1888.6 ± 14.8	1646.1 ± 33.3	<0.001	1851.7 (1546.3, 2200.9)	1772.2 (1460.2, 2137.1)	0.001
Daily protein intake, g/day	-	-	-	68.7 ± 0.7	57.1 ± 1.5	<0.001	62.6 (48.7, 78.0)	57.5 (44.7, 74.6)	<0.001
Daily fat intake, g/day	-	-	-	37.7 ± 0.6	27.3 ± 1.3	<0.001	30.0 (14.2, 41.2)	25.8 (17.0, 37.8)	<0.001
Daily CHO intake, g/day	-	-	-	311.7 ± 2.3	286.6 ± 5.6	<0.001	323.8 (278.1, 376.3)	315.9 (275.4, 375.6)	0.252
Skeletal muscle index	1.938 ± 0.324	1.530 ± 0.271	<0.001	0.768 ± 0.003	0.581 ± 0.007	<0.001	1.842 ± 0.309	1.519 ± 0.254	<0.001
NAFLD liver fat score	-	-	-	−1.381 ± 0.021	−0.9110 ± 0.064	<0.001	−1.958 ± 0.636	−1.747 ± 0.648	<0.001
NAFLD, %	37.1	66.5	<0.001	20.8	34.7	<0.001	-	-	-
History of CVD, %	4.2	7.8	<0.001	2.7	10.5	<0.001	2.1	3.1	0.081

Abbreviations: SMI, skeletal muscle mass index; LSMI, low skeletal muscle mass index; KNHANES, Korean National Health and Nutrition Examination Survey; KoGES, The Korean Genome and Epidemiology Study; LSMI, low skeletal muscle mass index; SE, standard error; BMI, body mass index; HDL, high-density lipoprotein; LDL, low-density lipoprotein; AST, aspartate aminotransferase; ALT, alanine aminotransferase; CHO, carbohydrate; NAFLD, non-alcoholic fatty liver disease; CVD, cardiovascular disease. * *p* derived from Student’s t-test for continuous variables and chi-square test for categorical variables. ^†^ *p* derived from weighted generalized linear regression analysis for continuous variables and weighted chi-square test for categorical variables.

**Table 3:** Relationship between NAFLD and LSMI status with or without abdominal obesity.

2016–2019 Gangnam Severance Hospital Check-Up	Normal SMI without Abdominal Obesity	LSMI without Abdominal Obesity	Normal SMI with Abdominal Obesity	LSMI with Abdominal Obesity	
		ORs (95% CIs)			*p*
Unadjusted	1 (reference)	2.57 (2.22–2.99)	7.16 (6.29–8.14)	9.22 (7.90–10.75)	<0.001
Model 1 *	1 (reference)	3.17 (2.70–3.73)	3.57 (3.08–4.14)	6.87 (5.85–8.07)	<0.001
Model 2	1 (reference)	2.76 (2.34–3.27)	3.14 (2.70–3.66)	5.67 (4.80–6.69)	<0.001
**2008–2010 KNHANES**	Normal SMI without abdominal obesity	LSMI without abdominal obesity	Normal SMI with abdominal obesity	LSMI with abdominal obesity	
		ORs (95% CIs)			*p*
Unadjusted	1 (reference)	1.78 (1.29–2.45)	3.90 (3.30–4.62)	4.89 (3.64–6.56)	<0.001
Model 1	1 (reference)	1.85 (1.24–2.75)	1.60 (1.25–2.06)	2.97 (2.12–4.18)	<0.001
Model 2	1 (reference)	1.67 (1.08–2.56)	1.42 (1.10–1.83)	2.63 (1.87–3.71)	<0.001
**KoGES: Ansan-Ansung study**	Normal SMI without abdominal obesity	LSMI without abdominal obesity	Normal SMI with abdominal obesity	LSMI with abdominal obesity	
		HRs (95% CIs)			*p*
Unadjusted	1 (reference)	1.60 (1.39–1.84)	2.15 (1.85–2.49)	3.19 (2.70–3.77)	<0.001
Model 1	1 (reference)	1.72 (1.48–2.00)	1.43 (1.21–1.70)	2.21 (1.84–2.67)	<0.001
Model 2	1 (reference)	1.57 (1.35–1.83)	1.39 (1.18–1.65)	2.15 (1.79–2.60)	<0.001

Abbreviations: HRs, hazard ratios; ORs, odds ratios; CIs, confidence intervals; LSMI, low skeletal muscle mass index; NAFLD, non-alcoholic fatty liver disease; KNHANES, Korean National Health and Nutrition Examination Survey; KoGES, Korean Genome and Epidemiology Study; CVD, cardiovascular disease. Model 1: Adjusted for age, sex, physical activity, smoking status, current drinking status, basal energy expenditure, and daily protein intake *. Model 2: Adjusted for variables in Model 1 plus mean blood pressure, fasting glucose, total cholesterol, and history of CVD. * daily protein intake was not adjusted in the analysis of the 2016–2019 Gangnam Severance Hospital Check-up data due to a lack of information.

## Data Availability

The KoGES dataset used in this study (Ansan-Ansung cohort) can be provided after review and evaluation of research plan by the Korea Centers for Disease Control and Prevention (http://www.cdc.go.kr/CDC/eng/main.jsp) (accessed on 1 February 2021). The KNANES dataset are publicly available through the KNHANES website (http://knhanes.cdc.go.kr) (accessed on 1 February 2021).

## Supplementary Materials

The following are available online at https://www.mdpi.com/2079-7737/10/2/122/s1, Table S1. Incidence of LSMI during the follow-up study, Table S2. Subgroup analysis of incident LSMI according to NAFLD status, Table S3, HR and 95% CI for incident LSMI per 1 SD increase in NAFLD-liver fat score, Table S4. Subgroup analysis of incident NAFLD according to LSMI status, Table S5. HR and 95% CI for incident NAFLD per 1 SD increase in SMI according to sex, Table S6. Definitions of previous risk models for NAFLD, Table S7. Equations to calculate basal energy expenditure (kcal/day), Figure S1. Kaplan-Meier curves for incident LSMI according to NAFLD status, Figure S2. Kaplan-Meier curves for incident NAFLD according to LSMI status, Figure S3. Flowchart of study population selection process in (a) KoGES, (b) KNHANES, and (c) GSHC.

## Abbreviations

NAFLD	Non-alcoholic fatty liver disease
WC	Waist circumference
BMI	Body mass index
MBP	Mean blood pressure
AST	Aspartate aminotransferase
ALT	Alanine aminotransferase
BEE	Basal energy expenditure
HDL	High-density lipoprotein
CVD	Cardiovascular disease
GSHC	Gangnam Severance Hospital Check-up
KNHANES	Korean National Health and Nutrition Examination Survey
KoGES	Korean Genome and Epidemiology Study
SMI	Skeletal muscle mass index
LSMI	Low skeletal muscle mass index
LDL	Low-density lipoprotein
HRs	Hazard ratios
CIs	Confidence intervals
SD	Standard deviations
ORs	Odds ratios
EI/BEE	Ratio of energy intake/basal energy expenditure
LECT2	Leukocyte cell-derived chemotaxin 2
HSP	Hepassocin
EGFR/JNK	Epidermal growth factor receptor/c-Jun N-terminal kinase
BIA	Bioelectrical impedance analysis
DXA	Dual energy X-ray absorptiometry
IRB	Institutional Review Boards
TSM	Total skeletal muscle mass
ASM	Appendicular skeletal muscle mass
FNIH	Foundation for the National Institutes of Health
SBP	Systolic blood pressure
DBP	Diastolic blood pressure
FFQ	Food frequency questionnaire
SE	Standard error
