# Supplementary material for: Relationship between Muscle Mass and Non-Alcoholic Fatty Liver Disease
Source: Biology (Basel). 2021 Feb 5;10(2):122. doi: 10.3390/biology10020122 (PMC7915258; doi:10.3390/biology10020122)
Supplement: Supplementary file 1 [file biology-10-00122-s001.zip › Figure S3. Flowchart of study population selection process in (a) KoGES, (b) KNHANES, and (c) GSHC.pptx]

## Slide 1
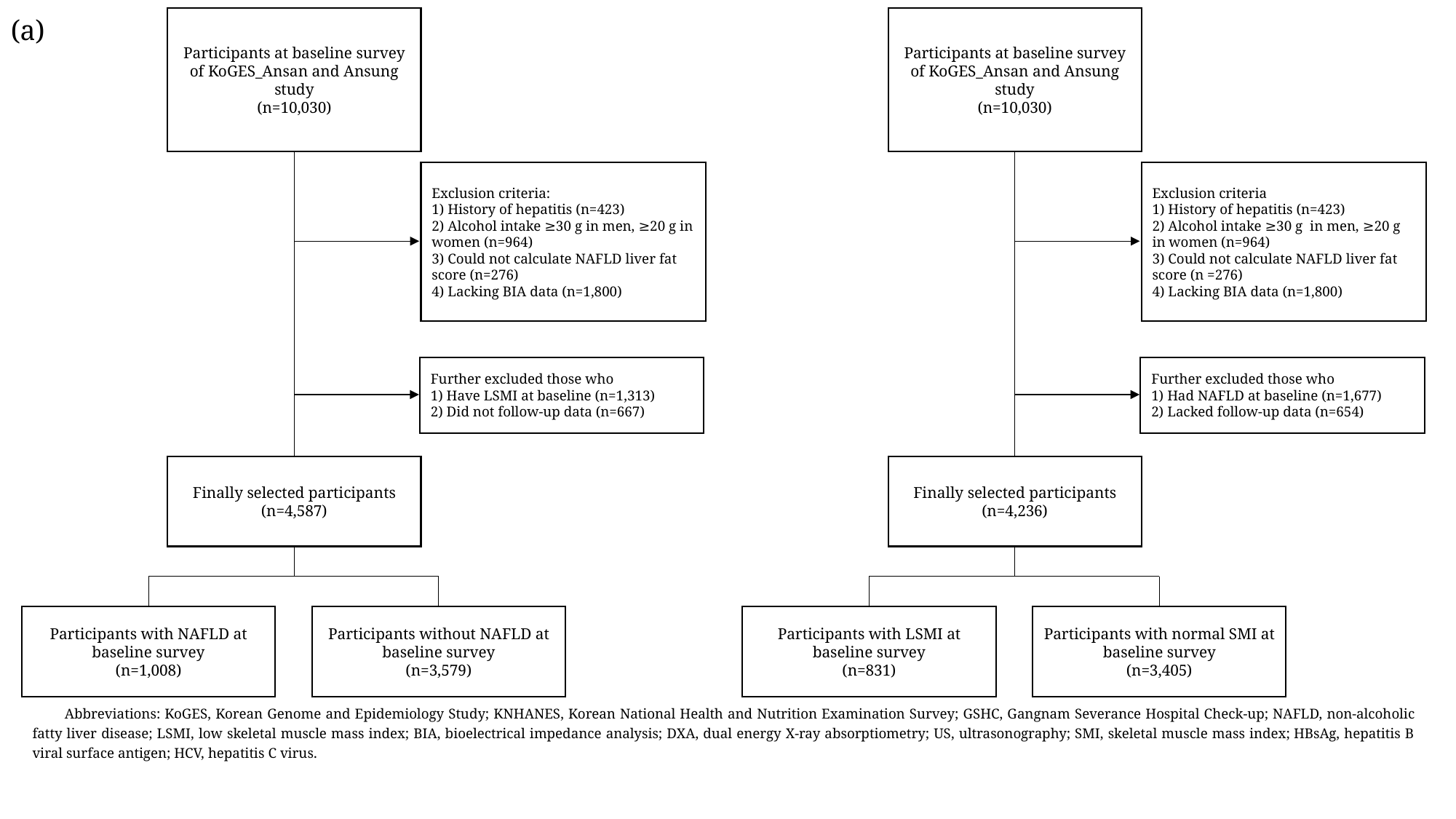

(a)
Participants at baseline survey of KoGES_Ansan and Ansung study
(n=10,030)
Exclusion criteria:
1) History of hepatitis (n=423)
2) Alcohol intake ≥30 g in men, ≥20 g in women (n=964)
3) Could not calculate NAFLD liver fat score (n=276)
4) Lacking BIA data (n=1,800)
Further excluded those who
1) Have LSMI at baseline (n=1,313)
2) Did not follow-up data (n=667)
Finally selected participants
(n=4,587)
Participants with NAFLD at baseline survey
(n=1,008)
Participants without NAFLD at baseline survey
(n=3,579)
Participants at baseline survey of KoGES_Ansan and Ansung study
(n=10,030)
Exclusion criteria
1) History of hepatitis (n=423)
2) Alcohol intake ≥30 g in men, ≥20 g in women (n=964)
3) Could not calculate NAFLD liver fat score (n =276)
4) Lacking BIA data (n=1,800)
Further excluded those who
1) Had NAFLD at baseline (n=1,677)
2) Lacked follow-up data (n=654)
Finally selected participants
(n=4,236)
Participants with LSMI at baseline survey
(n=831)
Participants with normal SMI at baseline survey
(n=3,405)
Abbreviations: KoGES, Korean Genome and Epidemiology Study; KNHANES, Korean National Health and Nutrition Examination Survey; GSHC, Gangnam Severance Hospital Check-up; NAFLD, non-alcoholic fatty liver disease; LSMI, low skeletal muscle mass index; BIA, bioelectrical impedance analysis; DXA, dual energy X-ray absorptiometry; US, ultrasonography; SMI, skeletal muscle mass index; HBsAg, hepatitis B viral surface antigen; HCV, hepatitis C virus.

## Slide 2
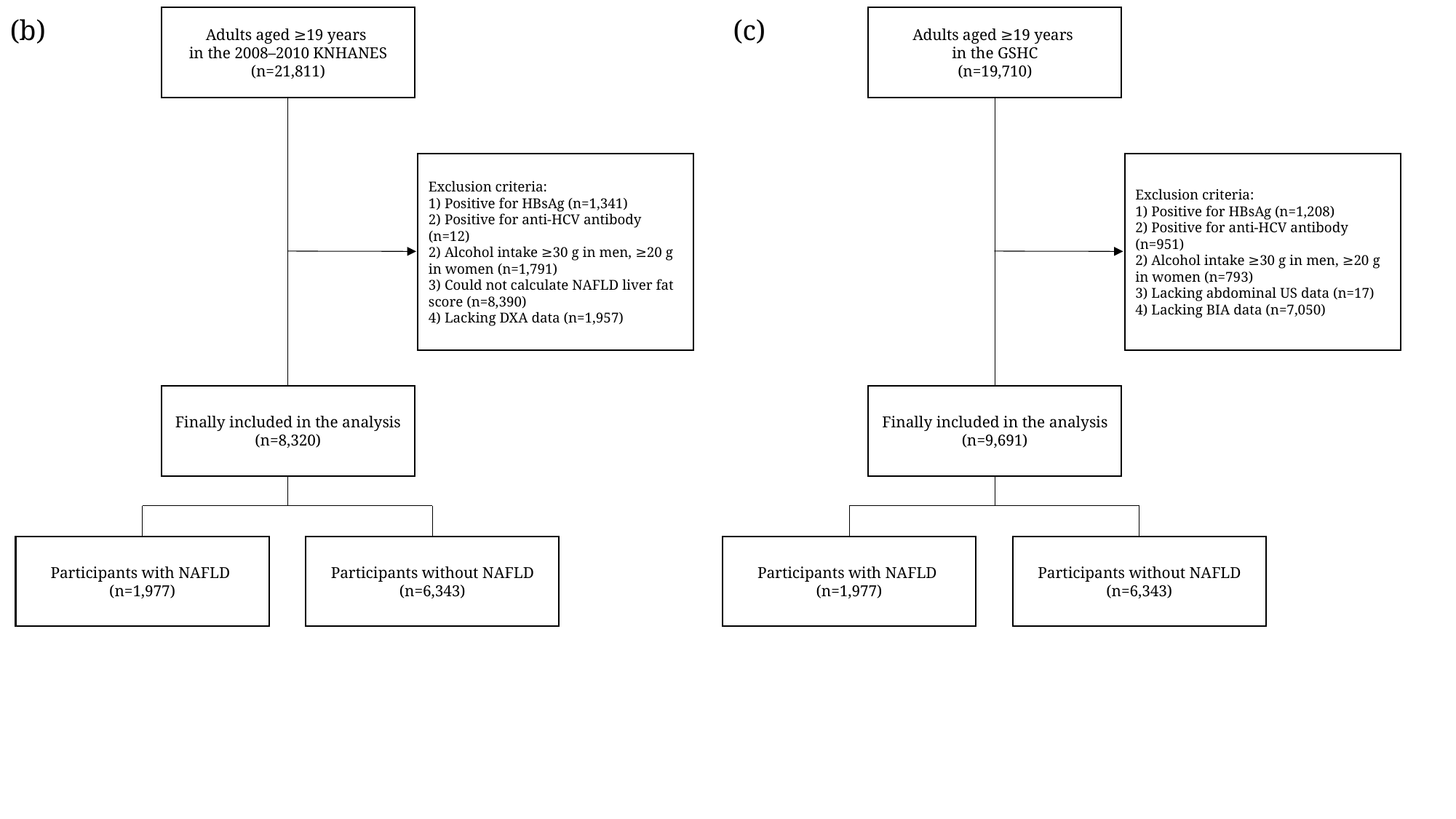

Adults aged ≥19 years
in the 2008–2010 KNHANES
(n=21,811)
Exclusion criteria:
1) Positive for HBsAg (n=1,341)
2) Positive for anti-HCV antibody (n=12)
2) Alcohol intake ≥30 g in men, ≥20 g in women (n=1,791)
3) Could not calculate NAFLD liver fat score (n=8,390)
4) Lacking DXA data (n=1,957)
Finally included in the analysis
(n=8,320)
Participants with NAFLD
(n=1,977)
Participants without NAFLD
(n=6,343)
Adults aged ≥19 years
in the GSHC
(n=19,710)
Exclusion criteria:
1) Positive for HBsAg (n=1,208)
2) Positive for anti-HCV antibody (n=951)
2) Alcohol intake ≥30 g in men, ≥20 g in women (n=793)
3) Lacking abdominal US data (n=17)
4) Lacking BIA data (n=7,050)
Finally included in the analysis
(n=9,691)
Participants with NAFLD
(n=1,977)
Participants without NAFLD
(n=6,343)
(c)
(b)
