# Supplementary material for: Relationship between Muscle Mass and Non-Alcoholic Fatty Liver Disease
Source: Biology (Basel). 2021 Feb 5;10(2):122. doi: 10.3390/biology10020122 (PMC7915258; doi:10.3390/biology10020122)
Supplement: Supplementary file 1 [file biology-10-00122-s001.zip › Supplementary Tables.docx]

**Table S1.** Incidence of LSMI during the follow-up study

| Year range | Follow-up | Incident NAFLD rate per 2 years | Incident LSMI rate per 2 years |
| --- | --- | --- | --- |
| 2001–2002 | Baseline |  |  |
| 2003–2004 | 2 years | 10.4 | 19.9 |
| 2005–2006 | 4 years | 8.6 | 8.0 |
| 2007–2008 | 6 years | 10.2 | 4.0 |
| 2009–2010 | 8 years | 9.3 | 3.0 |
| 2011–2012 | 10 years | 4.6 | 2.0 |
| 2013–2014 | 12 years | 5.0 | 2.7 |

Abbreviations: LSMI, low skeletal muscle mass index; NAFLD, non-alcoholic fatty liver disease.

**Table S2.** Subgroup analysis of incident LSMI according to NAFLD status

| **KoGES: Ansan-Ansung study** | Normal | NAFLD |  |  |
| --- | --- | --- | --- | --- |
| **Variables** |  | HRs (95% CIs) | *p* | interaction *p* |
| **Daily calorie intake** |  |  |  | 0. 228 |
| **High energy intake/BEE ratio** |  |  |  |  |
| Unadjusted | 1 (reference) | 1.58 (1.34–1.86) | <0.001 |  |
| Model 1 | 1 (reference) | 1.37 (1.136–1.66) | 0.001 |  |
| Model 2 | 1 (reference) | 1.27 (1.04–1.55) | 0.959 |  |
| **Low energy intake/BEE ratio** |  |  |  |  |
| Unadjusted | 1 (reference) | 1.49 (1.27–1.76) | <0.001 |  |
| Model 1 | 1 (reference) | 1.23 (1.01–1.50) | 0.039 |  |
| Model 2 | 1 (reference) | 1.15 (0.93–1.42) | 0.191 |  |
| **Physical activity** |  |  |  | 0.034 |
| **Yes** |  |  |  |  |
| Unadjusted | 1 (reference) | 1.47 (1.24**–**1.75) | <0.001 |  |
| Model 1 | 1 (reference) | 1.24 (1.01–1.52) | 0.036 |  |
| Model 2 | 1 (reference) | 1.14 (0.93–1.41) | 0.215 |  |
| **No** |  |  |  |  |
| Unadjusted | 1 (reference) | 1.51 (1.29–1.77) | <0.001 |  |
| Model 1 | 1 (reference) | 1.38 (1.14–1.66) | <0.001 |  |
| Model 2 | 1 (reference) | 1.28 (1.05**–**1.56) | 0.016 |  |

Model 1: Adjusted for age, sex, abdominal obesity, physical activity, smoking status, current drinking status, basal energy expenditure, and daily protein intake.

Model 2: Adjusted for variables in Model 1 plus the mean blood pressure, fasting glucose, total cholesterol, and history of CVD.

**Table S3**. HR and 95% CI for incident LSMI per 1 SD increase in NAFLD-liver fat score

|  |  | |  |  | |
| --- | --- | --- | --- | --- | --- |
| NAFLD-liver fat score per 1 SD increase | HR | 95% CI | | | *p* |
| Unadjusted | 1.18 | 1.13–1.22 | | | <0.001 |
| Model 1 | 1.16 | 1.12–1.21 | | | <0.001 |
| Model 2 | 1.16 | 1.10–1.21 | | | <0.001 |

Model 1: Adjusted for age, sex, abdominal obesity, physical activity, smoking status, current drinking status, basal energy expenditure, and daily protein intake.

Model 2: Adjusted for variables in Model 1 plus the mean blood pressure, fasting glucose, total cholesterol, and history of CVD.

Abbreviations: HR, hazard ratio; CI, confidence interval; LSMI, low skeletal muscle mass index; SD, standard deviation; NAFLD, non-alcoholic fatty liver disease; CVD, cardiovascular disease.

**Table S4.** Subgroup analysis of incident NAFLD according to LSMI status

| **KoGES: Ansan-Ansung study** | Normal | LSMI |  |  |
| --- | --- | --- | --- | --- |
| **Variables** |  | HRs (95% CIs) | *p* | interaction *p* |
| **Daily calorie intake** |  |  |  | 0.006 |
| **High energy intake/BEE ratio** |  |  |  |  |
| Unadjusted | 1 (reference) | 1.77 (1.49–2.09) | <0.001 |  |
| Model 1 | 1 (reference) | 1.69 (1.41–2.03) | <0.001 |  |
| Model 2 | 1 (reference) | 1.64 (1.37–1.97) | <0.001 |  |
| **Low energy intake/BEE ratio** |  |  |  |  |
| Unadjusted | 1 (reference) | 1.80 (1.54–2.10) | <0.001 |  |
| Model 1 | 1 (reference) | 1.63 (1.37–1.94) | <0.001 |  |
| Model 2 | 1 (reference) | 1.52 (1.27–1.82) | <0.001 |  |
| **Physical activity** |  |  |  | 0.503 |
| **Yes** |  |  |  |  |
| Unadjusted | 1 (reference) | 1.69 (1.43**–**1.99) | <0.001 |  |
| Model 1 | 1 (reference) | 1.57 (1.31–1.89) | <0.001 |  |
| Model 2 | 1 (reference) | 1.54 (1.29–1.85) | <0.001 |  |
| **No** |  |  |  |  |
| Unadjusted | 1 (reference) | 1.82 (1.55–2.14) | <0.001 |  |
| Model 1 | 1 (reference) | 1.72 (1.44–2.04) | <0.001 |  |
| Model 2 | 1 (reference) | 1.58 (1.33**–**1.89) | <0.001 |  |

Model 1: Adjusted for age, sex, abdominal obesity, physical activity, smoking status, current drinking status, basal energy expenditure, and daily protein intake.

Model 2: Adjusted for variables in Model 1 plus the mean blood pressure, fasting glucose, total cholesterol, and history of CVD.

**Table S5**. HR and 95% CI for incident NAFLD per 1 SD increase in SMI according to sex

|  | Men | |  |  | | |  | | Women | |  |  | |
| --- | --- | --- | --- | --- | --- | --- | --- | --- | --- | --- | --- | --- | --- |
| SMI per 1 SD increase | HR | 95% CI | | | *P* |  | | HR | | 95% CI | | | *p* |
| Unadjusted | 0.77 | 0.71–0.83 | | | <0.001 |  | | 0.65 | | 0.60–0.70 | | | <0.001 |
| Model 1 | 0.67 | 0.61–0.73 | | | <0.001 |  | | 0.70 | | 0.64–0.75 | | | <0.001 |
| Model 2 | 0.68 | 0.62–0.75 | | | <0.001 |  | | 0.72 | | 0.66–0.78 | | | <0.001 |

Model 1: Adjusted for age, sex, abdominal obesity, physical activity, smoking status, current drinking status, basal energy expenditure, and daily protein intake.

Model 2: Adjusted for variables in Model 1 plus the mean blood pressure, fasting glucose, total cholesterol, and history of CVD.

Abbreviations: HR, hazard ratio; CI, confidence interval; SD, standard deviation; SMI, skeletal muscle mass index; NAFLD, non-alcoholic fatty liver disease; CVD, cardiovascular disease.

**Table S6.** Definitions of previous risk models for NAFLD

| Model | Cut-off point | Equation |
| --- | --- | --- |
| NAFLD liver fat score | ≥ -0.640 | -2.89 + 1.18 × metabolic syndrome (yes = 1/no = 0) + 0.45 × diabetes (yes = 2/no = 0) + 0.15 × (fasting insulin, μU/L) + 0.04 × AST + 0.94 × AST/ALT ratio |

Abbreviations: NAFLD, non-alcoholic fatty liver disease; AST, aspartate aminotransferase; ALT, alanine transaminase

**Table S7.** Equations to calculate basal energy expenditure (kcal/day)

| Sex | Equations |
| --- | --- |
| Men | 204 - 4.00 * age(year) + 450.5 * height (m) + 11.69 * weight (kg) |
| Women | 255 - 2.35 * age(year) + 361.6 * height (m) + 9.39 * weight (kg) |
